# Vitamin D deficiency and visceral adipose tissue in early pregnant women

**DOI:** 10.1186/s12884-021-03888-1

**Published:** 2021-07-02

**Authors:** Rosangela Cisneiros, Juliana Segatto, Eloíse Paixão, Ítalo Bacellar, Marcelo Lima, Álvaro Pacheco, João Guilherme Alves, Francisco Bandeira

**Affiliations:** 1 Division of Endocrinology, University of Pernambuco Medical School, Recife, Brazil; 2 Medicine School, Universidade Federal do Vale do São Francisco, Avenida José de Sá Maniçoba, s/n, Centro, 56304-917 PE Petrolina, Brazil; 3 Division of Endocrinology and Radiology, Universidade Federal do Vale do São Francisco, Petrolina, Brazil; 4 Division of Pediatrics and Radiology, Instituto de Medicina Integral Prof. Fernando Figueira, Recife, Brazil

**Keywords:** Visceral adipose tissue, Vitamin D, Pregnancy, Ultrasound, Obesity

## Abstract

**Background:**

We aimed to assess the correlation between vitamin D serum level and visceral fat tissue during early pregnancy.

**Methods:**

This cross-sectional study was performed in Pernambuco, Brazil. 190 low risk pregnant women (8–16 gestational weeks) were eligible. Visceral adipose tissue was measured by ultrasonography following the technique described by Armellini. The 25(OH) D in serum was determined through chemiluminescence. The Spearman correlation test was applied to evaluate the correlation between vitamin D serum level and VAT, considering *p* < 0.05 to be significant.

**Results:**

Vitamin D insufficiency was present in 129 (67.8 %) of subjects. Pregnant women with or without vitamin D deficiency did not differ in age, gestational age, nutritional status and visceral adipose tissue. No correlation between visceral adipose tissue and 25(OH) D was observed: − 0.057 (*p* = 0.435).

**Conclusions:**

Maternal visceral adipose tissue and vitamin D serum level are not correlated during pregnancy.

## Background

Vitamin D deficiency (VDD) is a major global public health problem in all age groups, even in developed countries and regions with adequate ultraviolet radiation. Exposure to sunlight is the main source of vitamin D (VD) and factors such as season, time of day, latitude, skin phototype, sun exposure duration, type of clothing and the use of sunscreens may influence its synthesis [1–3]. Brazil, a tropical/subtropical country with elevated ultraviolet radiation has a very high prevalence of VDD [4]. VD has many actions in the body including the musculoskeletal system, calcium homoeostasis and immune system [5]. Recently, a link between VD and chronic non-communicable diseases has emerged including insulin resistance, diabetes and cardiovascular disease [6–8]. These chronic diseases are also commonly associated with obesity [9].

It is believed that VDD is associated with obesity, and adipose tissue may be responsible for its lower bioavailability. Visceral Adipose Tissue (VAT) easily absorbs VD by chemical affinity. Therefore, volumetric dilution has been proposed as the main mechanism to elucidate its low levels in obesity [10–12]. Two large epidemiological studies have shown that the thickness of VAT correlated inversely with the serum concentration of 25OHD in the adult population [11, 13]. Hao et al. found an inversely association between vitamin level and VAT in Chinese men [14]. Zhang et al. also reported a significant negative association between VAT and VDD in men and pre-menopausal women but not in post-menopausal women [15]. Batista et al. found that excess visceral adiposity, hypertriglyceridemia and high low-density lipoprotein cholesterol levels were strong predictors of hypovitaminosis D [16]. A meta-analysis of randomized clinical trials reported positive effects of VD supplementation on fat mass [17].

In pregnant women, VDD is also an important health problem with a prevalence ranging from 5 to 84 % [12, 18]. Besides, pregnancy is associated with a VAT increased and it has been linked to insulin resistance and hyperglycemia [19, 20]. However, we did not find in our search studies evaluating the association between VDD and VAT in pregnant women. We aimed to assess the correlation between VD serum level and VAT during early pregnancy.

## Methods

This cross-sectional study was performed at the Hospital Dom Malan located in Petrolina (9°S and 40°W), Pernambuco state, Northeast of Brazil. Hospital Dom Malan registers about 6 000 births per year. This region has an average ultraviolet index of 13 during most of the year.

Pregnant women aged 13–39 years and 8–16 weeks of gestational age were eligible. Exclusion criteria were twin pregnancy, diabetes mellitus, previous gestational diabetes or preeclampsia, fetal malformations, maternal mental disease. After screening, 190 pregnant women were included. All participants answered a questionnaire regarding socio-demographic and clinical information were taken from the medical record. Weight and height were measured according to the standardized methodology. Body mass index (BMI) was calculated by the formula: weight (kg) / height (m^2^) and Atalah classification was used [21]. VAT was measured by ultrasonography performed by a qualified specialist. A Philips D7 ultrasound device, equipped with a 3.0 to 7.0 MHz convex transducer, multifrequency (Bothell, WA/USA), was used. VAT was determined following the technique described by Armellini [22]: the convex transducer positioned immediately above the umbilical scar, the distance in centimeters being measured between the inner edge of the rectus abdominis muscle, at the point of its insertion in the alba line, and the anterior wall of the abdominal aorta.

The 25(OH) D in serum was determined through chemiluminescence, using the Atellica EVA-Siemens device (Erlangen/Germany) with a result expressed in nanograms per Milliliter. Insufficiency and deficiency values were respectively between 20 and 30 ng/mL and < 20 ng/mL, according to the values adopted by the Endocrine Society Practice Guidelines [23].

### Statistical analyses

Data were analyzed descriptively through absolute and percentage frequencies in the categorical variables and the measures: mean, standard deviation (mean ± SD), and median to numerical variables. To assess a significant association between two categorical variables, Pearson’s chi-square test including the likelihood ratio test and confidence interval (CI) was used. For the comparison between categories concerning the numerical variables, the Mann–Whitney test was used. To assess the degree of the relationship between two numerical variables, Spearman’s correlation coefficient and the specific Student’s t-test for the null correlation hypothesis were obtained.

The choice of the Mann–Whitney test and Spearman correlation was due to the absence of normality, which was verified by the Shapiro–Wilk test. The margin of error used in deciding the statistical tests was 5 and 95 % CI. The data were entered into the EXCEL spreadsheet, and the program used to obtain the statistical calculations was IMB SPSS in version 25.

## Results

190 pregnant women were studied. The mean age was 26 ± 5.76 years and pregnant women were included in the study with a 12.3 ± 2.5 weeks of gestation. The BMI ranged from 16.6 to 47.0 kg/m² (25.7 ± 4.9 kg/m²); 57 (30 %) were overweight and 27 (14.2 %) obese. 129 (67.8 %) of pregnant women had vitamin D insuficiency, i.e., serum value of 25 (OH) D < 30 ng/mL. The thickness of the VAT varied between 0.9 and 6.1 cm (2.9 ± 0.9 cm). Pregnant women with or without VDD did not differ in age, gestational age, nutritional status and VAT (Table 1). The Spearman value correlation between VAT and 25(OH) D was – 0.057, (*P value* = 0.435). Table 2 presents Spearman correlation between VAT, 25(OH) D and age, weight and BMI.

**Table 1:** Characteristics of pregnant women studied according to the vitamin D status

		**Vitamin D (30ng/mL)**														
**Variable**		**< 30**		**≥ 30**		**Total**					***P value***		**OR (CI 95 %)**			
		n	%	n	%	n			%							
**TOTAL**		**129**	**100.0**	**61**	**100.0**	**190**			**100.0**							
**Age range (years)**											*P* = 0.331					
13 a 19		11	8.5	11	18.0	22			11.6				1.00			
20 a 24		39	30.2	16	26.2	55			28.9				2.44 (0.88–6.75)			
25 a 29		44	34.1	16	26.2	60			31.6				2.75 (1.00–7.57)			
30 a 34		23	17.8	13	21.3	36			18.9				1.77 (0.60–5.20)			
35 a 42		12	9.3	5	8.2	17			8.9				2.40 (0.63–9.14)			
**Gestational age (weeks)**											*P* = 0.306					
< 10		16	12.4	4	6.6	20			10.5				1.72 (0.52–5.68)			
10–12		55	42.6	32	52.5	87			45.8				0.74 (0.39–1.41)			
> 12		58	45.0	25	41.0	83			43.7				1.00			
**Visceral adipose tissue (cm)**											*P* = 0.340					
< Percentile 25 (2.31)		30	23.3	18	29.5	48			25.3				1.00			
> Percentile 25 (2.31) to 50 (2.80)		36	27.9	13	21.3	49			25.8				1.66 (0.70–3.94)			
> Percentile 50 (2.80) to 75 (3.50)		30	23.3	19	31.1	49			25.8				0.95 (0.42–2.15)			
> Percentile 75 (3.50)		33	25.6	11	18.0	44			23.2				1.80 (0.73–4.42)			
**ATALAH**											*P* = 0.838					
Low weight		12	9.3	5	8.2	17			8.9				1.00			
Normal weight		59	45.7	32	52.5	91			47.9				0.77 (0.25–2.37)			
Overweight		39	30.2	17	27.9	56			29.5				0.96 (0.29–3.14)			
Obesity		19	14.7	7	11.5	26			13.7				1.13 (0.29–4.39)			
**Variable**		**Vitamin D (30ng/mL)** **< 30 (*n* = 129)** Median ± SD (Average)							**≥ 30 (*n* = 61)** **Median ± SD (Average)**				***P* value**			
Pre gestational weight		65.03 ± 13.08 (64.00)							62.07 ± 11.93 (58.00)				***P* = 0.108**			
Weight		66.33 ± 13.32 (63.70)							63.73 ± 12.25 (61.30)				***P* = 0.196**			
Body Mass Index		25.96 ± 5.07 (24.96)							25.27 ± 4.69 (24.88)				***P* = 0.343**			

**Table 2:** Spearman correlation between visceral adipose, 25(OH) D and age, weight and body mass index

Variable	Visceral adipose tissue	25(OH)D
Age	0,299 (< 0,001)^a^	-0,117 (0,108)
Weight	0,525 (< 0,001)^a^	-0,111 (0,126)
Body Mass Index	0,574 (< 0,001)^a^	-0,130 (0,074)

(^1^)Statistically different from zero

## Discussion

In the present study we did not find association between serum VD values and visceral fat in pregnant women. For the best of our knowledge, it is the first time that this association is assessed during pregnancy. Some studies have described an inverse association between VDD and visceral fat in non-pregnancy populations [24, 14]. However, among the various physiological changes that occur during pregnancy, the redistribution of adipose tissue is one of them. Although not yet properly studied, it is admitted that an increase in visceral adipose tissue occurs, but the specific function of visceral adipose in pregnancy is still unknown. Thus, it seems likely that visceral adipose tissue has somewhat distinct functions in pregnancy and our findings cannot be compared with non-pregnant populations.

Recently, Carreras-Badosa et al. found that maternal serum VD was inversely associated with visceral fat and in their offspring at the age of 5–6 years [25]. However, diet and lifestyle habits were not studied in both mothers and their children, and this, rather than maternal VD status could explain the relationship between lower maternal serum VD and offspring adiposity.

An increased risk of VDD has been described worldwide in obese individual. The underlying explanations are not clear. Receptors of VD are expressed in adipose tissue and 25(OH) D could modulate adipogenesis through VD receptors-dependent inhibition of specific components [26]. Other explanation is based on experimental findings that deficiency of VD can increases lipogenesis upregulating adipocyte calcium signaling and improving the secretion of parathyroid hormone [27]. However, the role of VD in adipogenesis or other functions of VAT during pregnancy is still unknown.

Pregnancy VDD has been described with a prevalence of 18–84 % [9]. In the present study, the prevalence of VDD was high (68 %). This value was similar to other Brazilian studies and higher than that described in countries such as Spain and United States [28]. Brazil is a tropical country with continental dimensions and abundant sunshine. The municipality of Petrolina is located at approximately − 9°, which favors the synthesis of VD. However, there is a paradox in countries with low solar incidence and with a lower frequency of VDD when compared with countries with higher solar incidence, certainly because of protection measures that prevent its synthesis and bioavailability [29].

In the multicenter HAPO study, increased maternal BMI was associated with lower maternal 25(OH) D levels [30]. However, BMI is not an accurate measure of fat tissue, especially during pregnancy. Besides, the deposition of adipose tissue preferably occurs in two distinct anatomical places, VAT and subcutaneous adipose tissue, and BMI does not differentiate them. We determine VAT through ultrasound and this method has been shown to be safe, effective, simple and reproducible [31].

Our study has some limitations. At first, a cross-sectional design was performed and we could not determine the causal relationship between VD and VAT. Second, other possible important variables confounding the VD status, like sun light exposition, dietary habits and possible seasonal variations in VD, were not evaluated in our study. However, despite these limitations our study has strengths. The present study is the first to assess the correlation of VD with VAT thickness in pregnant women. A large sample was studied and specific methods to measure VD and VAT were used.

## Conclusions

Our data demonstrated that maternal visceral adiposity and low concentrations of VD were not associated during pregnancy. However, as this was the first study to assess the association between VAT and VD in pregnant women, further studies are needed to confirm these findings.

## Data Availability

The datasets used and/or analysed during the current study are available from the corresponding author on reasonable request.

## Abbreviations

VD: Vitamin D
VDD: Vitamin D Deficiency
VAT: Visceral Adipose Tissue
BMI: Body Mass Index
